# Phlegmonous gastritis as the initial presentation of acute myeloid leukaemia

**DOI:** 10.1002/jha2.462

**Published:** 2022-04-28

**Authors:** Shin Lee, Kei Fujita, Moe Kato, Hiroyuki Makino, Takeshi Hara, Hisashi Tsurumi

**Affiliations:** ^1^ Department of Hematology and Oncology Matsunami General Hospital Gifu Japan; ^2^ Department of Internal Medicine Matsunami General Hospital Gifu Japan

**Keywords:** AML, CT image, phlegmonous gastritis

1

A 74‐year‐old Japanese man who had received one cycle of azacitidine for myelodysplastic syndrome and regularly used proton pump inhibitors was hospitalised with sudden‐onset stomach ache and fever. He had been administrated meropenem and micafungin for febrile neutropenia during the azacitidine therapy until 3 days before admission. Clinical laboratory data on admission showed: white blood cell count, 0.7 × 10^9^/L (neutrophils 33.4%, blasts 1.0%); haemoglobin, 10.2 g/dl; platelet count, 84 × 10^9^/L; and C‐reactive protein, 32.7 mg/dl. Computed tomography revealed diffuse oedematous thickening of the whole gastric wall, despite the absence of duodenum obstruction and a small amount of ascites (Figure 1). He had started immediate empirical administration of meropenem and micafungin because of the neutropenia and remained nil‐by‐mouth after admission. Bone marrow aspiration revealed an increase in myeloid blasts (41.5%), and acute myeloid leukaemia (AML) was diagnosed. Upper gastrointestinal endoscopy on day 2 of hospitalisation showed oedematous thickening of the folds of the whole gastric body and multiple ulcers with irregular margins from the corpus of the stomach to the fornix (Figure 1). Gastric mucosal biopsy was performed due to the possibility of leukaemia involvement. Fever and stomach ache persisted despite 5 days of antibiotic therapy, so low‐dose cytarabine (20 mg/body/day) and etoposide (50 mg/body/day) were administrated intravenously for 10 days as induction therapy. Gastric mucosal biopsy showed necrotic tissue with numerous internal bacterial masses. No leukaemic blasts showing CD34 or KIT positivity were identified, and a diagnosis of phlegmonous gastritis (PG) was finally made. Blood and gastric biopsy tissue cultures yielded negative results. The patient showed gradual clinical improvement under continued antibiotic therapy during chemotherapy. On follow‐up 5 weeks later, repeated upper gastrointestinal endoscopy showed improvement of the endoscopic findings. The patient made a complete recovery with no relapse.

**FIGURE 1:**
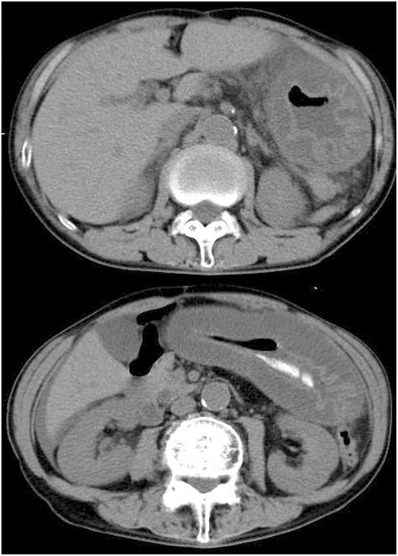
Images of abdominal computed tomography and upper gastrointestinal endoscopy

PG is a rare entity as a differential diagnosis of acute abdomen and often lethal acute infection of the stomach. Prompt antibiotic therapy based on early diagnosis is required. As mucosal injury due to gastric cancer, peptic ulcer or endoscopic procedure is regarded as the main cause of PG, few cases of PG with haematological malignancies have been reported. Even in this patient who was regularly using proton pump inhibitors and had no history of endoscopic manipulation, the initial onset of AML can on rare occasions cause fatal infection such as PG due to neutropenia. As with our patient, a rare but potentially fatal gastric infection can be an initial feature of AML, due to the severe neutropenia resulting from AML, despite the continuous use of proton pump inhibitors and the lack of prior endoscopic manipulation.

## FUNDING INFORMATION

No funding was recieved.

## CONFLICT OF INTEREST

The authors declare that they have no conflict of interest.

## AUTHOR CONTRIBUTIONS

SL, KF and HT contributed to the conception and the design of the study. SL contributed to the literature review and the writing of the manuscript. MK, HM, TH, and HT contributed to the revision of the manuscript. KF contributed to figure creation. SL and KF confirm the authenticity of all the raw data. All authors approved of the final manuscript version submitted for publication.

## ETHICS STATEMENT

In accordance with our institution's ethics board, written consent forms are not required as less than 10 cases are reported.

